# Exploring Sex and Clinical Factors Associated with Long-Term Survival After Implantable Cardioverter-Defibrillator Implantation: A 10-Year Cohort Study

**DOI:** 10.3390/jcm15031275

**Published:** 2026-02-05

**Authors:** Rebeca Lorca, María Salgado, Cristina Helguera, Alberto Alen, Francisco González-Urbistondo, Rosana González-Mesa, Paula Bouzón, Daniel García, Rodrigo Di Massa, Rut Álvarez-Velasco, José Manuel Rubín, Pablo Avanzas

**Affiliations:** 1 Área del Corazón, Hospital Universitario Central Asturias, 33011 Oviedo, Spain; msalgadobarq@gmail.com (M.S.); cristina.h.amezua@gmail.com (C.H.); albertoalen@usal.es (A.A.); frangonzalez_95@hotmail.com (F.G.-U.); rosana.glez.mesa@gmail.com (R.G.-M.); paulabouzoniglesias@gmail.com (P.B.); danigr1985@gmail.com (D.G.); radm1982@gmail.com (R.D.M.); jmrl100@gmail.com (J.M.R.); avanzas@gmail.com (P.A.); 2 Unidad de Cardiopatías Familiares, Área del Corazón y Departamento de Genética Molecular, Hospital Universitario Central Asturias, 33011 Oviedo, Spain; 3 Departamento de Medicina, Universidad de Oviedo, 33003 Oviedo, Spain; 4 Instituto de Investigación Sanitaria del Principado de Asturias (ISPA), 33011 Oviedo, Spain; 5 Redes de Investigación Cooperativa Orientadas a Resultados en Salud (RICORs), 28029 Madrid, Spain; 6 Centro de Investigación en Red de Enfermedades Cardiovasculares (CIBERCV), 28029 Madrid, Spain

**Keywords:** sex differences, ICD, survival analysis

## Abstract

**Background**: Sex-related differences in outcomes after implantable cardioverter-defibrillator (ICD) implantation remain incompletely understood. Although women receive ICDs less frequently, whether their long-term survival differs from that of men in real-world clinical practice is not well established. We aimed to evaluate sex-specific mortality and relative survival in a large consecutive cohort of ICD recipients from a tertiary hospital. **Methods**: We conducted a retrospective cohort study including all consecutive patients who underwent ICD implantation at a tertiary hospital between 2015 and 2025. Demographic features, device indication, and mortality were obtained through clinical records. Relative survival (observed vs. expected) was estimated using the Ederer II method with national life tables. A Cox proportional hazards model assessed the effect of sex on mortality. **Results**: A total of 1091 patients (82.1% men; mean age 63.1 ± 13.1 years) were included. During a mean follow-up of 4.33 ± 3.22 years, 230 patients died (21.1%). Women showed lower unadjusted all-cause mortality than men: 24 deaths (18.0%) vs. 206 (20.6%). Women had significantly higher left ventricular ejection fraction (41.5 ± 23.6% vs. 37.2 ± 18.1%, *p* = 0.0046), less ischemic cardiomyopathy, and lower prevalence of cardiovascular risk factors. Although univariable analysis suggested lower mortality in women (HR 0.58, 95% CI 0.38–0.90; *p* = 0.014), multivariable analysis indicated that sex was not an independent predictor of mortality (HR 0.81, 95% CI 0.53–1.26). Relative survival revealed a substantial long-term mortality burden in ICD carriers, especially in men: men: 4-year survival 82.3% (expected 93.2%); 8-year 66.7% (85.6%); 12-year 56.0% (76.8%); women: 4-year survival 89.1% (expected 96.7%); 8-year 77.1% (92.8%); 12-year 77.1% (89.2%). **Conclusions**: In this large real-world cohort of ICD recipients, women showed lower unadjusted mortality and a smaller excess mortality compared with the general population. However, sex was not an independent predictor of survival after multivariable adjustment. These findings may indicate that observed survival differences are largely explained by differences in clinical profile and comorbidity burden rather than by sex itself.

## 1. Introduction

Implantable cardioverter-defibrillators (ICDs) are a cornerstone therapy for preventing sudden cardiac death (SCD) in patients at high arrhythmic risk. The ESC (European Society of Cardiology) Guidelines [1] recommend ICD for primary prevention in patients with ischemic dilated cardiomyopathy (DCM) in NYHA class I and left ventricular ejection fraction (LVEF) ≤30% despite ≥3 months of optimal medical therapy (OMT) (class IIa); or NYHA class II-III and LVEF) ≤35% despite ≥3 months of OMT (class I). The evidence for ICD benefit in non-ischemic DCM patients is slightly weaker. Risk stratification is in non-ischemic DCM and other cardiomyopathies or channelopathies are more individualized, often based on family history, syncope, NSVT, or genetic findings [1,2].

Despite robust evidence from randomized trials, real-world cohorts often differ in age, comorbidities, and underlying cardiomyopathy. In particular, sex disparities are remarkable, as women are consistently under-represented in trials [3,4,5] and they receive ICDs less frequently than men [6,7,8], even when guideline eligible.

Although the prognostic impact of sex after ICD implantation remains controversial, sex-related differences have been described in association with the development and progression of heart failure [3,9]. Some studies suggest women experience fewer ventricular arrhythmias and better survival [10,11,12,13], while others report no differences between sexes [14,15,16]. Additionally, relative survival, comparing outcomes to the general population matched by age and sex, has barely been explored in ICD cohorts.

The objectives of this study were therefore to evaluate sex-specific mortality and relative survival in a large consecutive cohort of ICD recipients from a tertiary hospital, and to compare outcomes with the corresponding general reference population.

## 2. Materials and Methods

### 2.1. Study Design and Population

We conducted a retrospective, observational cohort study including all consecutive adult patients who underwent implantation of an ICD, either alone or in combination with cardiac resynchronization therapy (CRT-ICD), at the Hospital Universitario Central de Asturias (HUCA), a tertiary referral center, between January 2015 and December 2024. In the Principality of Asturias, healthcare coverage is universal and access to the public health system is guaranteed for all residents, independent of their socioeconomic or demographic characteristics. Specialized care for ICD implantation is territorially organized into virtual catchment areas, whereby patients are referred according to their place of residence either to HUCA (Area IV/central-southwest Asturias) or to Hospital Universitario de Cabueñes (Area V/eastern-coastal Asturias), an administrative division aimed solely at optimizing care pathways and response times, with both university hospitals providing comparable services to mixed urban and rural populations. Consequently, the consecutively enrolled patients in this study represent the entire, unselected population of ICD recipients from the HUCA catchment area and are representative of the general population living in this part of Asturias, neutralizing the risk of selection bias related to access to medical care.

ICD indication was given by the cardiologist-assisting clinician at the main hospital and only referred to the reference center for ICD implantation.

The cohort included both primary and secondary prevention indications, irrespective of underlying cardiac disease. Patients younger than 14 years or without available follow-up data were excluded.

Follow-up duration was recorded for all patients, and no significant differences were observed between device types (ICD vs. CRT-ICD) or between indications (primary vs. secondary prevention), ensuring comparability across subgroups.

### 2.2. Data Collection

Clinical and device data were extracted from electronic health records and device interrogation reports. Variables extracted and used in analyses included: age at implantation, sex, left ventricular ejection fraction (LVEF), presence of severe systolic dysfunction (<35% LVEF), underlying cardiac diagnosis (detailed categories), cardiovascular risk factors (CVRF) like hypertension, diabetes, dyslipidemia, active smoking and alcohol use, ICD indication (primary/secondary prevention), device type (single-chamber ICD, dual-chamber ICD, CRT-ICD, subcutaneous ICD), follow-up time since implantation, ICD therapies (appropriate and inappropriate), type of ventricular arrhythmia treated, arrhythmic storm, and vital status with cause of death classification.

We constructed the control reference population using mortality tables provided by the National Institute of Statistics of Spain (Instituto National de Estadística [INE]). Extensive information about mortality data stratified by age ranges, sex, and autonomous regions in Spain (including Asturias) is accessible and publicly available on its official website. To compare our sample of probands with ICD with the control cohort, we matched our population data with the mortality data provided in the public tables published by the INE with participants of the same age, sex, geographical area, and year of the event. All patients were followed for at least 1 year, and this study ended in January 2025.

This study was approved by the institutional local Ethical Committee (CEImPA 2025.108) and conformed to the principles of the Declaration of Helsinki. Given the retrospective nature of this study, informed consent was waived.

### 2.3. Study Endpoints

The primary endpoint was to compare the differences in all-cause mortality analyzing differences between sexes. Secondary outcomes included the comparison of survival rates with those in the general population of the same age and geographical region.

### 2.4. Statistical Analysis

Continuous variables were expressed as mean ± standard deviation. Categorical variables were expressed as absolute numbers and percentages. Between-group differences were analyzed using Student’s *t*-test or χ^2^ test as appropriate. Kaplan–Meier curves were generated to compare survival between men and women, with statistical significance assessed using the log-rank test. Sex-specific mortality risk was assessed using univariable Cox proportional hazards regression, reporting hazard ratios (HRs) with 95% confidence intervals (CIs). The predictive capacity of the model was evaluated with the Harrel c test and survival was assessed using Kaplan–Meier curves. A *p*-value < 0.05 was considered statistically significant.

Relative survival was calculated using the Ederer II method, comparing the observed survival of ICD carriers to the expected survival of the general population matched by: age, sex, and calendar year. Relative survival was estimated at 4, 8, and 12 years for each sex. The following concepts were defined as reported elsewhere [17]:

1. Observed survival (OS): This was considered as the probability of surviving all causes of death in the group of ICD patients. It was represented with a CI of 95% and estimated using the life table method.
2. Expected survival (ES): This variable represents the expected mortality of the general population of the same age and geographical area, the control cohort from Asturias. All data were obtained from publicly reported data by the INE. As ES had no sampling error, confidence intervals were not estimated [18].

Recently, specially supported by cancer registries and strong studies, Ederer II is becoming the method of choice to evaluate survival, especially when the follow-up time is less than 10 years, as other methods are considered to overestimate survival [19,20]. Given the mean follow-up time of our cohort, we selected the Ederer II method for this study. As a result, we calculated ES with the Ederer II method, which considers individuals at risk until the corresponding patient dies [21]. The Ederer II method works under the assumption that when ES is modeled, deaths from a specific cause like ICD contribute a negligible proportion of all the deaths in the reference population [22]. When the ES is included within the 95% CI of the OS, it is considered that there are no differences between the ES and the OS.

3. Relative survival (RS): RS is a ratio calculated from the OS of our ICD population during a specific time interval to the ES of the control cohort [21,23,24].
4. Excess mortality (EM): This refers to the percentage of the mortality in probands with ICD that is exclusively attributable to the cardiovascular disease that led to ICD implantation. It is obtained by subtracting 1 minus RS. Consequently, if the EM was 0%, that would mean that none of the ICD patients died due to cardiovascular disease. Accordingly, if the 95% CI included the number 0, it means that no statistically significant differences were found, and that there was no evidence to support otherwise.

All statistical analysis was performed using STATA/IC 15.1 (StataCorp LLC, College Station, TX, USA). The “strs” command by Dickman et al. [21] was used to estimate the OS, ES, and RS.

## 3. Results

Among the 1091 ICD recipients, 897 were men (82.1%) and 194 were women (17.8%). The mean age at implantation was 63.1 ± 13.1 years, without significant sex differences (*p* = 0.271). Baseline characteristics can be consulted in Table 1.

Women presented with better systolic function, with higher LVEF (41.5% vs. 37.2%, *p* = 0.0046) and less frequently severe LV dysfunction (46.7% vs. 54.5%, *p* = 0.0465). The underlying etiology also differed substantially by sex (*p* < 0.001). Men predominantly exhibited ischemic cardiomyopathy (53.9%), while women showed higher proportions of non-ischemic cardiomyopathy (39.0%) and hypertrophic cardiomyopathy (12.1%). Cardiovascular risk factors were significantly more prevalent in men, including hypertension, diabetes, dyslipidemia, smoking, and alcohol use (*p* < 0.05). ICD indication (primary vs. secondary prevention) did not differ by sex. Women received proportionally more CRT-ICD and subcutaneous devices (*p* < 0.001). The mean follow-up was 4.33 ± 3.22 years, similar in both groups.

Over follow-up, 20.6% of patients received appropriate ICD therapy and 7.0% experienced inappropriate shocks, with no sex differences. Ventricular tachycardia accounted for most treated arrhythmias (61.7%). Arrhythmic storm occurred in 1.83% of the cohort without differences by sex.

### Mortality, Survival and Sex Effect

**Figure 1:**
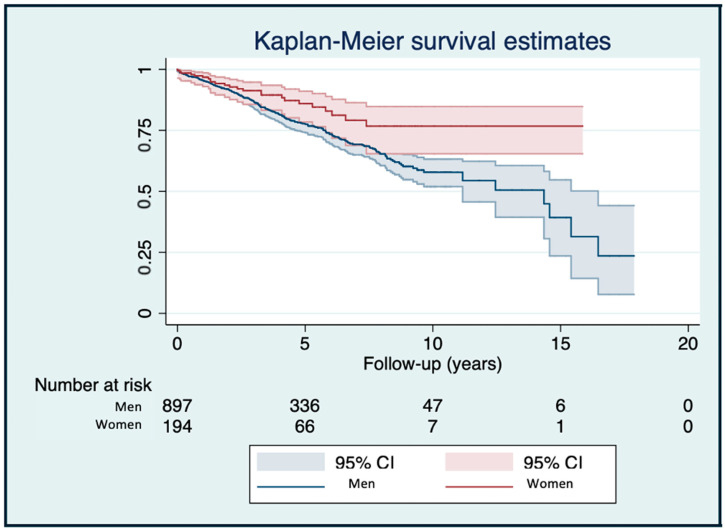
Kaplan–Meier survival analysis comparing men and women with implantable cardioverter-defibrillator.

A total of 230 deaths (21.1%) occurred. In unadjusted analyses, mortality was higher in men than in women (22.97% vs. 12.31%, *p* = 0.001). Causes of death were predominantly non-cardiac (35.2%) or unknown (43.9%, Table 1). Univariable Cox regression showed female sex was associated with a lower risk of death (HR 0.58 (95% CI 0.38–0.90), *p* = 0.014, Figure 1. However, multivariable Cox regression (adjusted by age, LVEF, ischemic vs. non-ischemic etiology, cardiovascular risk factors, device type and ICD indication—primary vs. secondary) was non-significant (Table 2).

**Figure 2:**
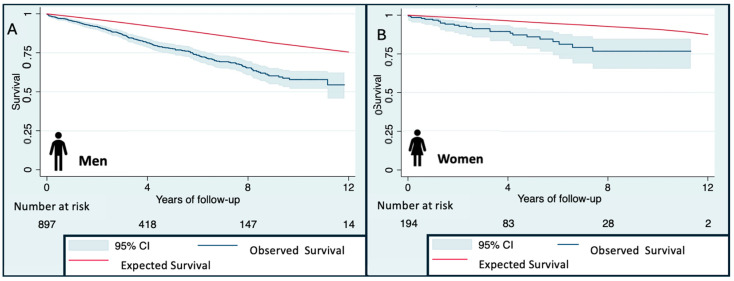
Gender differences in survival analysis of 1091 patients with the same access to the medical care system who were referred by their cardiologist-assisting clinician to the reference center for ICD implantation, when compared with the reference population.

Analysis of observed survival, expected survival, and excess mortality showed statistically significant worse outcomes in patients with ICD, when compared with the reference general population. However, relative survival consistently favored women, with higher observed survival at 4, 8, and 12 years compared with men (Figure 2, Table 3), reflecting differences in overall mortality patterns rather than an independent effect of sex.

## 4. Discussion

The present study, analyzing a large real-world cohort of 1091 patients who underwent ICD implantation over a 10-year period, found that crude long-term outcomes differed by sex, with women showing lower unadjusted all-cause mortality. However, this association was not independent after multivariable adjustment. This likely reflects differences in baseline clinical characteristics, as women had a substantially lower burden of CVRF and better LVEF at baseline compared with men. In the adjusted model, sex was not an independent predictor of survival (HR 0.81; 95% CI 0.53–1.26). Furthermore, relative survival (observed vs. expected) revealed a smaller survival gap for women compared with the general population than for men, likely reflecting differences in baseline risk and competing mortality rather than sex-specific ICD benefit.

In this context, our findings should also be interpreted alongside international data. Population-based studies from the United States have shown a marked decline in DCM-related mortality over the past two decades, coinciding with broader uptake of implantable cardioverter-defibrillators and contemporary heart failure therapies [25,26,27]. However, men with ICD due to DCM consistently had 2- to 2.5-fold higher age-adjusted mortality rates compared with women [27]. Our results extend these observations to a broader ICD population by highlighting that, even within a contemporary European cohort with universal healthcare access, differences in long-term survival patterns by sex persist after ICD implantation, largely reflecting disparities in baseline clinical profiles and comorbidity burden rather than differential benefit from ICD therapy.

Our findings are consistent with previous studies reporting lower unadjusted mortality and arrhythmic burden in women after ICD implantation, although these associations are variably attenuated after adjustment for clinical confounders. For example, Saxena et al. reported that women exhibited a significantly lower risk of both first and recurrent ventricular tachyarrhythmias compared to men across ischemic and non-ischemic cardiomyopathy, with the protective effect being particularly pronounced in the non-ischemic subgroup [28]. A meta-analysis of gender differences in ICD outcomes including 46,657 patients and reporting multivariable adjusted gender-specific hazard ratio, found that women had a lower risk of all-cause mortality compared with men in adjusted analyses [10], although the magnitude and independence of this association varied across studies. Similarly, van der Lingen et al. reported that in a cohort of 2300 patients, male ICD patients were at higher risk of death and device therapy than females, regardless of ischemic versus non-ischemic substrate [14]. A German registry showed that female patients had a higher risk of major periprocedural and in-hospital complications but a lower all-cause and cardiovascular mortality [29].

By contrast, earlier investigations had been inconclusive. For example, Burger et al. found no significant difference in overall survival between sexes in an Austrian cohort of 1471 ICD recipients, though women received fewer appropriate antitachycardia pacing therapies [30]. As previously advanced, the heterogeneity of ICD populations, differing clinical practices and under-representation of women likely may have contributed to the inconsistent findings [3,4,5,6,7,8]. In this scenario, our study adds to the literature by providing a large contemporary cohort with long follow-up and detailed baseline phenotyping, reinforcing the finding that favorable outcomes observed in women with ICDs are primarily associated with differences in clinical profile and disease characteristics rather than sex itself as an independent prognostic factor.

Several factors may contribute to the observed differences in survival patterns between women and men in this study:

1. Lower burden of comorbidities and risk factors. In our cohort women had significantly lower prevalence of hypertension, diabetes, dyslipidemia, smoking and alcohol use—recognized contributors to both arrhythmic and non-arrhythmic mortality.
2. Less advanced myocardial dysfunction. Women exhibited a higher mean LVEF (41.5% vs. 37.2%, *p* = 0.0046) and a lower proportion of severe dysfunction (46.7% vs. 54.5%, *p* = 0.0465), suggesting they were implanted at an earlier phase of disease or had less structural damage.
3. Different etiologic substrate. The etiology of cardiomyopathy differed significantly by sex (*p* < 0.001). Men predominantly had ischemic cardiomyopathy, which is associated with larger scar burden, greater arrhythmic risk and higher competing mortality. Women had higher proportions of non-ischemic cardiomyopathy and hypertrophic cardiomyopathy, whose arrhythmic risk may evolve differently and whose response to ICD therapy and heart failure therapies may be more favorable.
4. Arrhythmic risk and therapy burden. Several studies suggest that women may have a lower incidence of ventricular tachyarrhythmias and may receive fewer appropriate therapy deliveries. For example, Saxena et al. found that women had almost half the risk of recurrent VTA events compared with men [28]. Lower arrhythmic burden may translate into fewer ICD shocks or therapies, which themselves have been associated with adverse outcomes.
5. Biological sex differences in myocardial and electrophysiological substrate. Emerging data suggest sex hormones, myocardial fibrosis patterns, and ion channel differences may influence arrhythmia susceptibility and remodeling. For instance, younger pre-menopausal women appear to have less myocardial fibrosis and more favorable diastolic properties. Although not directly measured in our cohort, these mechanisms may contribute to the observed differences between sexes.
6. Device selection and therapy management. Although our study did not demonstrate major sex differences in inappropriate therapy (6.70% women vs. 7.02% men) or appropriate therapy (18.04% women vs. 20.62% men, *p* = 0.416), it is conceivable that differences in referral, device programming or follow-up may influence outcomes. Prior research suggests women may receive different device types or display different responses to CRT [31,32]. There is a well-described sex disparity in ICD implantation rates, not fully explained by epidemiological differences in the prevalence of cardiomyopathies, which could imply undertreatment of women [31]. Women may differ from men in baseline characteristics at implantation, suggesting a selection bias [31].

Importantly, interpretation of sex-related differences must be tempered by uncertainty regarding causes of death. Approximately 44% of deaths in our cohort were classified as of unknown cause, and over one-third were clearly non-cardiac. This substantially limits conclusions regarding arrhythmic versus non-arrhythmic mortality and precludes definitive attribution of the observed survival advantage to ICD-mediated prevention of sudden cardiac death. Consequently, our findings should be interpreted as reflecting overall prognosis after ICD implantation rather than ICD-specific arrhythmic benefit.

The use of relative survival analysis also warrants careful interpretation. Relative survival estimates excess mortality compared with the general population and reflects the global burden of cardiovascular disease, comorbidities and frailty in ICD recipients, rather than the performance or failure of ICD therapy itself. Therefore, the observed survival gap—particularly pronounced in men—should not be interpreted as reduced ICD efficacy, but rather as a marker of higher competing non-arrhythmic mortality and overall disease severity. Although women demonstrated a smaller excess mortality compared with the general population, it remains uncertain whether these sex differences in relative survival would persist after full adjustment for age, baseline health status and comorbidity burden.

### 4.1. Guidelines and Implications for Practice

Current clinical practice guidelines recognize ICD implantation for both primary and secondary prevention of sudden cardiac death (SCD). The 2022 European Society of Cardiology (ESC) Guidelines for the management of patients with ventricular arrhythmias and prevention of SCD recommend ICD implantation class I for secondary prevention in survivors of sudden cardiac arrest or sustained ventricular arrhythmias, and in selected primary prevention patients (e.g., LVEF ≤ 35%, symptomatic heart failure, optimal medical therapy [1]. The 2017 American College of Cardiology/American Heart Association/Heart Rhythm Society (ACC/AHA/HRS) Guideline similarly emphasizes that candidates for ICD implantation should have a reasonable expectation of survival with good functional status for ≥1 year [33]. These guidelines do not currently emphasize sex as a formal variable in implantation decision pathways and our findings do not support the use of sex as an independent variable for risk stratification or clinical decision-making after ICD implantation.

In particular, the observed lower unadjusted mortality in women indicates that concerns about under-referral of women for ICD implantation cannot be justified on the basis of worse outcomes. Studies have shown that women are less likely to receive ICDs even when eligible: Ingelaere et al. noted significant sex bias in ICD implantation practices [31].

From a practical standpoint, clinicians should ensure that female patients with guideline indications for ICD are not overlooked. Moreover, clinicians should recognize that all patients, particularly male patients and those with ischemic cardiomyopathy and a high burden of CVRF, may warrant enhanced surveillance, optimization of heart failure therapies and risk factor modification. Finally, future guideline updates or registry analyses may further explore whether sex modifies risk in conjunction with established clinical variables, without supporting sex-specific thresholds in isolation. Evaluating whether sex should be incorporated as part of the risk stratification process alongside other established variables (e.g., LVEF, heart failure class, etiology, comorbidities) is a future challenge.

This study was conducted in a Spanish tertiary hospital within a universal healthcare system, which minimizes access-related disparities in ICD implantation, follow-up and device management. While this context strengthens internal validity, it may limit external generalizability to healthcare systems without universal coverage, where socioeconomic factors, insurance status and referral patterns may play a greater role in ICD utilization and outcomes. Additionally, ICD implantation practices, programming strategies and patient selection may vary across regions. Therefore, caution is warranted when extrapolating our findings to health systems with different organizational or reimbursement structures. Nonetheless, the consistency of our results with prior international registries supports the broader relevance of sex-based differences in ICD outcomes.

### 4.2. Limitations and Future Directions

While the present study offers important insights, several limitations merit consideration. As a single-center retrospective cohort, there is potential for selection bias and unmeasured confounding (e.g., myocardial scar burden, detailed imaging markers, device programming differences). The multivariable adjustment incorporating all relevant confounders (age, LVEF, CVRF, etiology, device type and indication) included in the present analysis did not show sex as an independent predictor. Other parameters were not evaluated in this multivariable adjustment. Moreover, a considerable proportion of deaths (≈44%) were classified as unknown cause, limiting inferences regarding arrhythmic vs. non-arrhythmic mortality. This high proportion of deaths with unknown cause introduces uncertainty in distinguishing arrhythmic from non-arrhythmic mortality and limits ICD-specific mechanistic interpretation. As a result, the observed sex differences should be viewed primarily as differences in overall survival patterns rather than definitive evidence of differential protection against sudden cardiac death. Finally, although relative survival analysis provides a valuable framework to contextualize observed mortality relative to the general population, it does not fully account for individual-level differences in baseline health status beyond age and sex. Residual confounding is therefore possible, and future studies using matched population controls, competing-risk approaches or multivariable-adjusted relative survival models are warranted to further elucidate sex-specific prognostic trajectories in ICD recipients.

## 5. Conclusions

In this large real-world cohort of ICD recipients, women exhibited lower crude all-cause mortality and a smaller excess mortality compared with the general population. However, sex was not an independent predictor of survival after multivariable adjustment. These findings may suggest that sex-related differences in prognosis are largely driven by baseline risk profiles and comorbidities, and do not support the use of sex alone as a determinant for risk stratification or follow-up strategies in ICD patients.

## Figures and Tables

**Table 1:** Baseline characteristics.

Variable	Total (n = 1091)	Men (n = 897)	Women (n = 194)	*p*-Value
Age at implantation, years (mean ± SD)	63.07 ± 13.12	63.27 ± 12.80	62.13 ± 17.32	0.271
LVEF, % (mean ± SD)	37.93 ± 19.25	37.16 ± 18.12	41.50 ± 23.58	0.0046
Severe LVEF dysfunction, n (%)	580 (53.41%)	489 (54.52%)	91 (46.67%)	0.0465
Underlying cardiomyopathy, n (%)				
- Ischemic cardiomyopathy	588 (53.90%)	519 (57.86%)	69 (35.79%)	<0.001
- Non-ischemic DCM	332 (30.43%)	258 (28.76%)	74 (38.95%)	
- Hypertrophic cardiomyopathy	76 (6.97%)	53 (5.91%)	23 (12.11%)	
- Non-dilated left ventricular cardiomyopathy, arrhythmogenic right ventricular cardiomyopathy or restrictive cardiomyopathy	17 (1.56%)	14 (1.56%)	3 (1.54%)	
- Primary arrythmia (without channelopathy or cardiomyopathy diagnosis)	17 (1.56%)	10 (1.11%)	7 (3.16%)	
- Brugada syndrome	38 (3.48%)	31 (3.46%)	7 (3.68%)	
- Long QT syndrome	18 (1.65%)	9 (1.00%)	9 (3.68%)	
- Short QT syndrome	1 (0.09%)	1 (0.11%)	-	
- Myotonic dystrophy type 1	2 (0.18%)	1 (0.11%)	1 (0.05%)	
- Inflammatory cardiac diseases	2 (0.18%)	1(0.11%)	1 (0.05%)	
Cardiovascular risk factors, n (%)				
- Hypertension	599 (54.90%)	508 (55.63%)	91 (46.91%)	0.014
- Diabetes mellitus	345 (31.62%)	297 (33.11%)	48 (24.74%)	0.023
- Dyslipidemia	587 (53.80%)	501 (55.85%)	86 (44.33%)	0.004
- Smoker	676 (61.96%)	596 (66.44%)	80 (41.24%)	<0.001
- Alcohol use	230 (21.08%)	221 (24.64%)	9 (4.64%)	<0.001
ICD indication, n (%)				
- Primary prevention	822 (75.55%)	666 (74.50%)	156 (80.41%)	0.082
- Secondary prevention	266 (24.45%)	228 (25.50%)	38 (19.59%)	
Device type, n (%)				
- Single-chamber ICD	756 (69.49%)	641 (71.70%)	115 (59.28%)	<0.001
- Dual-chamber ICD	53 (4.87%)	43 (4.81%)	10 (5.15%)	
- CRT-ICD	231 (21.23%)	177 (19.80%)	54 (27.84%)	
- Subcutaneous ICD	48 (4.41%)	33 (3.69%)	15 (7.73%)	
Follow-up, years (mean ± SD)	4.33 ± 3.22	4.39 ± 3.26	4.06 ± 3.02	0.194
ICD therapy, n (%)				
- Inappropriate therapy	76 (6.97%)	63 (7.02%)	13 (6.70%)	0.873
- Appropriate therapy	220 (20.60%)	185 (20.62%)	35 (18.04%)	0.416
Arrhythmic storm	20 (1.83%)	18 (2.01%)	2 (1.03%)	0.358
All-cause mortality	230 (21.06%)	206 (22.97%)	24 (12.31%)	0.001
- Terminal Heart Failure	14.78% (34)	14.56% (30)	16.67% (4)	
- Electrical storm	5.65% (13)	5.34% (11)	8.33% (2)	
- Endocarditis	0.43% (1)	0.43% (1)	-	
- Not cardiological	35.22% (81)	36.41% (75)	25.00% (6)	
- Unknown	43.91% (101)	43.20% (89)	50.00% (12)	

DCM: dilated cardiomyopathy, LVEF: left ventricular ejection fraction; ICD: implantable cardioverter-defibrillator; CRT: cardiac resynchronization therapy; SD: standard derivation.

**Table 2:** Cox regression survival analysis, univariable and multivariable.

Variable	Univariable Hazard Ratio (95% CI)	Multivariable Hazard Ratio (95% CI)
**Sex**	0.58 (0.38–0.89)	0.81 (0.53–1.26)
**Age**	1.06 (1.04–1.07)	1.05 (1.04–1.07)
Severe LVEF dysfunction	1.96 (1.49–2.57)	1.63 (1.21–2.19)
Ischemic cardiomyopathy vs. non-ischemic etiologies	2.76 (2.03–3.74)	1.62 (1.17–2.25)
**CVRF**		
Hypertension	1.96 (1.48–2.60)	1.25 (0.92–1.69)
Dyslipidemia	1.68 (1.29–2.21)	1.01 (0.75–1.35)
Diabetes mellitus	1.83 (1.41–2.38)	1.33 (1.01–1.74)
Smoking	1.63 (1.23–2.18)	1.33 (0.98–1.80)
**Device type**		
Single-chamber ICD	REF.	REF.
Dual-chamber ICD	0.42 (0.17–1.01)	0.51 (0.21–1.24)
CRT-ICD	0.93 (0.65–1.31)	0.69 (0.48–0.99)
Subcutaneous ICD	0.09 (0.01–0.64)	0.31 (0.04–2.25)
**ICD indication** (primary vs. secondary indication)	0.82 (0.70–0.96)	1.04 (0.75–1.43)

CI: confidence interval; ICD: implantable cardioverter-defibrillator; LVEF: left ventricular ejection fraction; CVRF: cardiovascular risk factors.

**Table 3:** Observed and expected survival male and female patients with implantable cardioverter-defibrillator, compared to the reference population. Excess mortality by 5-year annual intervals was also calculated. The data are expressed as a percentage (95% CI).

Years of Follow-Up	Cumulative Survival ICD Recipients (Observed Survival)	Expected Survival
**Male Patients with ICD**		
**4 years**	82.26% (95% CI 79.27–84.86%)	93.15
**8 years**	66.70% (95% CI 62.28–70.74%)	85.62
**12 years**	56.03% (95% CI 49.56–62.00%)	76.82
**Female Patients with ICD**		
**4 years**	89.08% (95% CI 82.79–93.16%)	96.66
**8 years**	77.10% (95% CI 66.71–84.62%)	92.85
**12 years**	77.10% (95% CI 66.71–84.62%)	89.21

ICD: implantable cardioverter-defibrillator.

## Data Availability

The data presented in this study are available on reasonable request from the corresponding author due to privacy and ethical restrictions, and subject to institutional approval.
